# Assessing the Need for Reproductive Genetic Counseling Among Adults with Cystic Fibrosis

**DOI:** 10.21203/rs.3.rs-9227324/v1

**Published:** 2026-05-11

**Authors:** Ellis Coulter, Sierra Knapp, Maddy Grassy, Rebecca Mueller, Christopher H. Goss, Tijana Milinic, Kathleen J. Ramos, Siddhartha G. Kapnadak

**Affiliations:** University of Washington; University of Washington; University of Washington; University of Pennsylvania; University of Washington; University of Washington; University of Washington; University of Washington

**Keywords:** Genetic counseling, cystic fibrosis, family planning, reproductive, CFTR modulators

## Abstract

**Background:**

Reproductive health has become an increasingly important focus for people with cystic fibrosis (CF), and guidelines have recommended routine inclusion of genetic counselors on care teams to address questions regarding infertility, pregnancy planning, prenatal diagnosis, and other reproductive health considerations. Despite this, implementation of genetic counseling is still lacking, and there is limited literature documenting patient perspectives on reproductive genetic counseling, particularly in the era of highly effective cystic fibrosis transmembrane conductance regulator (CFTR) modulators that have transformed the landscape of clinical care.

**Methods:**

An electronic REDCap survey was distributed to adults with CF at our center to assess the need for reproductive genetic counseling and to understand current reproductive health concerns. Descriptive statistics were used to report quantitative and demographic data, and a thematic analysis was performed for free-response data.

**Results:**

A total of 237 patients were surveyed, and 116 (49%) responded. Of the 116 respondents, 31 (27%) had never heard of genetic counseling, and 82 (71%) had not previously received it. Forty-four of these 82 (54%) reported that they would find genetic counseling moderately or very helpful. The most important reproductive health topics included the effect of CFTR modulators on fertility and pregnancy, and carrier screening for partners and family members. Qualitative analysis revealed key content categories, including *fertility status*, *concerns and uncertainty with personal health*, and *lack of informed discussions with providers*. Multiple respondents also highlighted *not wanting to pass CF onto a child*.

**Conclusions:**

Our findings highlight a need and desire in the modern era among adults with CF for improved education regarding reproductive health.

## Background

As people with cystic fibrosis (CF) live longer, healthier lives, reproductive health has become an increasingly important focus. Over 95% of males with CF are born with congenital bilateral absence of the vas deferens (CBAVD), causing obstructive infertility (1). Between 24–35% of females with CF also experience infertility and subfertility due to several factors including thickening of cervical epithelial mucus impacting sperm penetration, recurrent infections, inflammation, diabetes, and malnutrition (2). Due to these fertility barriers, many people with CF (pwCF) could potentially benefit from assisted reproductive technologies (ART), including in vitro fertilization, sperm retrieval, gamete cryopreservation, or intrauterine insemination. However, despite the availability of reproductive technologies, some pwCF may not be aware of their options or lack access to informed family planning (3).

Genetic counselors are health care providers who specialize in genetic disorders, aid in reproductive planning and decision making, and provide patient education and psychosocial counseling (4). Genetic counselors also coordinate genetic testing, which could be useful for pwCF who are interested in reproductive services including carrier screening for their partners, preimplantation genetic testing, and prenatal diagnosis. These considerations are increasingly important in the era of highly effective cystic fibrosis transmembrane conductance regulator (CFTR) modulators as life expectancy rises, more pregnancies occur, and the effects of modulator use during and after pregnancy continue to be explored (3, 5, 6). Although incorporating genetic counseling into adult CF care was recently recommended (6), availability and access to counselors can be limited for pwCF (7, 8). Moreover, it is currently unclear what specific genetic counseling needs are most important to pwCF and how to best deliver these services. Notably, the availability of CFTR modulators has certainly created more opportunities for reproduction, but whether patient perspectives have evolved in the modern era of CF therapeutics has also not been investigated thoroughly. The objective of this study was to identify sexual and reproductive health (SRH) knowledge gaps and describe genetic counseling needs in a contemporary cohort of adults with CF.

## Methods

The study was conducted at a single, adult CF center. During the study period, individuals did not routinely have access to a genetic counselor within the CF center. However, those who were interested in family planning were referred to reproductive specialists outside of the CF center, including within the institution’s maternal-fetal medicine clinic. The study was approved by our center’s institutional review board (STUDY 22709).

An electronic REDCap survey (see supplement) was created by two CF pulmonologists and a genetic counseling trainee, with the advice of a licensed reproductive genetic counselor. The survey collected demographic information, opinions on which SRH topics are most important to pwCF, and qualitative data from free-response questions. The survey was disseminated to adults (≥ 18 years) with CF at our center through a quarterly newsletter and via informational handouts that were distributed in person and over email from April through July 2025. Because the survey and study materials were in English, non-English speaking individuals were excluded from the study. Informed consent was obtained from all participants in the study.

Survey responses were reported using descriptive statistics. Five-point Likert scales were used to assess respondents’ opinions on the helpfulness of genetic counseling and various SRH topics. Participants’ responses to the two open-ended questions were exported from REDCap into excel and analyzed using inductive content analysis (9). Four team members familiarized themselves with the open-ended responses and collaboratively developed codes. Team members assigned codes to all textual responses in Excel and met regularly to compare coding and organize codes into content categories. The study team included individuals with expertise in CF, genetic counseling, and qualitative research, and included a researcher who also has CF.

## Results

A total of 237 adults at our center were contacted, of whom 116 (49%) completed the survey. Median age was 35 years (range 19–84 years), 48 (41%) of respondents were assigned male at birth, and 113 (97%) reported White race. CFTR modulators were used by 107 (92%) of respondents, with most on elexacaftor/tezacaftor/ivacaftor (Table 1).

Of the 116 respondents, 37 (32%) reported being parents of at least one child. Twenty-eight (24%) reported planning on having children in the future, 65 (56%) reported no plans, while 23 (20%) reported being undecided about their future reproductive plans. A majority (64%) of participants reported that their CF diagnosis influences their decision to have children.

### Experiences and opinions on genetic counseling

**Figure 1:**
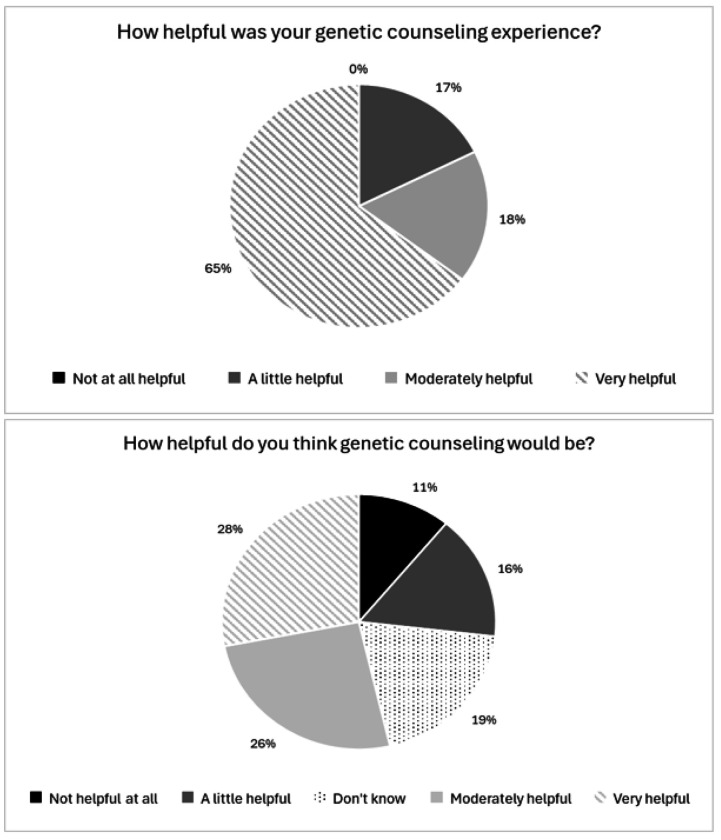
Perceptions of Genetic Counseling among Adults with Cystic Fibrosis. A) Responses to question regarding helpfulness of prior genetic counseling experiences. B) Responses to question regarding how helpful future genetic counseling would be.

Prior to the survey, 85 (73%) respondents had heard of genetic counseling, and 32 (29%) had personally received it. For those who had received genetic counseling, the majority (65%) reported their experience as “very helpful,” 18% reported it as “moderately helpful,” and none reported that it was “not at all helpful” (Figure 1A).

The 82 (71%) respondents who had not previously received genetic counseling were surveyed on how helpful they might expect it to be (with genetic counseling defined as “a process of helping people understand the medical, psychosocial, and familial implications of genetic disorders, like CF”) (4). Responses are shown in Figure 1B, with 61 (54%) reporting that genetic counseling would be “moderately” or “very helpful,” and only 9 (11%) reported that it would be “not at all helpful.” Of the latter group, eight of nine individuals reported that they do not plan to have children, while one reported being undecided. There was no relationship between age and perceived helpfulness of future genetic counseling.

**Figure 2:**
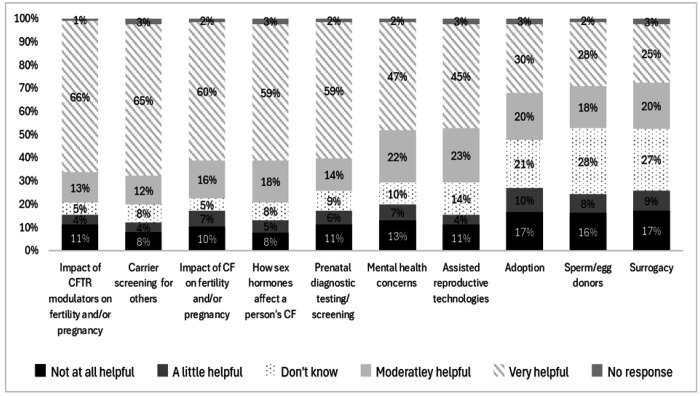
Importance of Different Sexual and Reproductive Health Topics Ten different SRH topics rated on a Likert scale of importance, ordered from highest proportion of “very helpful” responses selected to lowest.

Participants were also asked to rate a list of SRH learning priorities using a Likert scale (Figure 2). The topics of highest importance were “the impact of CFTR modulators on pregnancy and/or fertility,” “carrier screening for partners and family members,” and “the impact of CF on fertility or pregnancy,” with 66%, 65%, and 60% of respondents, respectively, rating these as “very helpful” topics (Figure 2). General SRH concerns, such as “how sex hormones including oral contraceptives influence a person’s CF,” were also rated “very helpful” by most participants and appeared in the qualitative analysis as well.

### Qualitative analysis

Responses to the two short response questions: “Does your diagnosis of CF impact your desire to have children?” and “What reproductive health concerns do you have?” were analyzed together given significant overlap in responses. Nine main content categories with corresponding subcategories were identified (Table 3), as detailed below.

#### Fertility status

Many pwCF noted having some knowledge about (male) infertility due to CBAVD and female subfertility, but others expressed uncertainty regarding fertility status, with one stating “I’ve never had my sperm tested, so I’m not sure if I even am fertile.” (ID# 121, male) and another asking “(am I) even able to get pregnant?” (ID# 058, female). Also apparent was the relationship between infertility and an individual’s well-being. One participant described feeling remorse about their inability to have children due to their CF, explaining “I had incredible shame that ‘I was the one at fault’ for not conceiving. Our marriage was falling apart at the time also. I did not connect the problems in the marriage with the grief I was experiencing at not conceiving” (ID# 034, female).

##### Concerns and uncertainty with personal health:

Participants described different concerns and uncertainty with their personal health, with responses spanning CF-related health problems, prenatal/pregnancy-associated concerns, and postnatal/parental-health concerns. Many noted being concerned about their ability to carry a pregnancy safely, or that having children could put their own health at risk. Some specifically mentioned that risks and uncertainty may still be present even with new treatments in the modern era of CF care: “Even on modulators, I need more sleep, and have other chronic conditions that are, if not caused by CF, exacerbated by CF,” one participant noted (ID# 056, female).

##### Ability to be present for children:

The concept of personal health also extended to one’s ability to be present for children, which was a common consideration that impacted some individuals’ decision to have children. One participant noted “I don’t know how I would be able to responsibly care for a child and take care of my own health” (ID# 056, female). Others reflected on the potential for limited life expectancy and noted that the ability to be present was a key responsibility of parenthood not only to a child, but also to one’s partner (Table 3). Although some felt that non-biologic parenting may be an option, for others the concept of one’s own risk for early mortality also applied to adoption: “Regarding adoption, (I) was concerned I would not live long enough to fully raise adopted child to adulthood,” explained one participant (ID# 073, male).

#### Concerns about children’s health/not wanting to pass on CF

In addition to the relationships between CF, one’s own health, and parenthood, participants describing having concerns about their potential children’s health and not wanting to pass on CF. Participants emphasized the mental and physical difficulty of living with CF and not wanting their children to experience the same health complications. One participant even noted that they did not want to create more CF carriers. Not surprisingly, partner carrier screening was an important family planning subcategory, with one respondent elaborating, “my having CF does affect WHO I will have another kid with and/or HOW they would be conceived. I want my partner to be tested for the CF gene first, as I would like to avoid sticking my child with that burden” (ID# 096, female).

#### Financial considerations with family planning

Another content category that intersected with reproductive decision-making was financial considerations with family planning. Financial concerns spanned the costs of reproductive technologies, to raising a child while also managing one’s CF, to the notion of uncertainty and insecurity associated with financial constraints. One participant noted “I have concerns about the financial strain-living with CF is already expensive, and raising a child adds another layer of uncertainty.” (ID# 027, female).

#### Advancements in treatments create reproductive opportunities

A few participants mentioned that improved health due to new therapies had created reproductive opportunities. As one participant explained, “Now with the new modifiers available to the CF community, I think I would have had children” (ID# 063, male). Respondents additionally commented on a lack of knowledge regarding the relationship between new CF treatments and various aspects of family planning.

##### Late diagnosis:

The content category of late diagnosis also intersected with fertility and reproductive health. In one case, a participant explained how infertility might have been explained by (at that time) undiagnosed CF: “My wife and I did IVF and sperm retrieval because I found out that it was my only option to try to have kids, and wasn’t diagnosed with CF until 10 + years later” (ID# 084, male). A few others mentioned they were diagnosed after reproductive age, so CF was not a factor in the decision for them to have children.

#### General/other reproductive health considerations

Participants also commented on a variety of additional SRH concerns which were categorized into a general/other reproductive health considerations category including questions about menopause, hormones, and birth control. Some wondered how CF affects their menopausal timeline, while others had concerns about which birth control options might be best for them in the context of having CF. For example, one participant noted “no one told me the depo shot causes decreased bone density” (ID# 039, female). Both males and females also conveyed a need for more CF-specific information on SRH, along with a need to advocate for oneself to acquire more knowledge.

#### Lack of informed discussions with providers

Lastly, despite the complex SRH categories and needs noted above, several participants emphasized a lack of informed discussions with providers during CF clinic as well as visits with primary care providers and gynecologists.

## Discussion

This survey study of a relatively large contemporary cohort of adults with CF, yielded several important findings regarding SRH and genetic counseling. First, although guidelines suggest routine inclusion of genetic counselors on CF care teams (5, 6), this was an uncommonly used service at our CF center during the study period. In fact, nearly 30% of our cohort had never heard of genetic counseling prior to the study, and less than 30% reported having personally received genetic counseling. Despite this, most respondents did indicate that genetic counseling was or would be helpful, and several specific topics were identified as important SRH educational opportunities, including how CF and CFTR modulators influence pregnancy and fertility, services such as carrier screening and prenatal diagnosis, assisted reproductive technologies, and the relationship between SRH and mental health. A major recurring concept was uncertainty among people with CF regarding SRH, with many respondents reporting a lack of clarity regarding fertility status, the relationship between CF and pregnancy, and how hormones and hormonal contraceptives may impact health, while also noting the difficulties obtaining proper education on these topics from their CF and other providers.

Outcomes in CF have improved dramatically with new therapies including highly effective CFTR modulators. As pwCF live longer, healthier lives in the modern era, the importance of SRH and family planning has increased substantially. Unfortunately, prior studies have shown significant SRH knowledge and educational gaps for pwCF (10). In one study, Kazmerski et al. showed that only 55% of CF care providers felt comfortable addressing SRH for women with CF, and only 51% believed they knew how to respond to a patient’s SRH concerns (11). Hailey et al. similarly showed that many pwCF had only limited or no discussions about SRH with their CF providers, and instead they sought out SRH advice from online forums (12). Another very recent qualitative study by Rajanikanth et al. showed that adults with CF understood the genetics of CF and the importance of genetic counseling, but only half had received genetic counseling before (7). Our study demonstrated a similar interest and need for reproductive genetic counseling, while also adding detailed perspectives from pwCF regarding a wide variety of SRH concerns and knowledge gaps. Genetic counseling was generally felt to be a helpful resource but not routinely used, and survey responses conveyed that pwCF were unable to have informed discussions regarding SRH and family planning with their care teams, including CF providers, primary care, and even women’s health specialists. These gaps in SRH discussions leave the potential for many knowledge voids, which were expressed by participants in many ways, including both uncertainty and adverse impact on mental health.

An important concept identified in our contemporary cohort was the multi-directional interaction of one’s CF health with pregnancy and parenthood. Concerns were broad and included their ability to carry a pregnancy safely (as it relates to their CF health), the risks to their own health from having a young child, and their ability to be present for their child (if their CF health deteriorates). One prevalent content category also related to CF health was the fear of “passing on” CF. Respondents acknowledged the difficulties of a life with CF, and some even felt a sense of “responsibility” to avoid passing the condition to offspring. These considerations are important and potentially an opportunity for education, particularly with the availability of CF carrier screening, as well as other technologies in the modern era, including preimplantation genetic testing (PGT-M) embryo screening and selection/in vitro fertilization, or prenatal diagnosis and termination. While many barriers exist and may be more pronounced in resource-limited settings including costs, time, availability of genetic counseling, and conflict with personal values or laws, SRH and family planning education certainly appears to be a potential area for improvement in the modern era of CF care. An online manual on SRH targeted to people with CF and CF clinicians was developed to guide clinical conversations about related topics and may provide a starting place for such initiatives (13).

Importantly, 92% (107 participants) of our study population were taking CFTR modulator therapy at the time of surveys. Recent literature shows improvement in female fertility while using modulators and an increase in the number of pregnancies in women with CF since the availability of elexacaftor/tezacaftor/ivacaftor in particular (3, 14, 15). In our cohort, about one third had previously had children, while over half were either planning or undecided about having children in the future. While the impact of CFTR modulators on fertility is clear, there is limited literature documenting whether and how patient perspectives on SRH in the modulator era have evolved. On one hand, improvements in overall health with CFTR modulators could be seen as having the potential to reduce the complexity of family planning, but our data do not support that notion. Most respondents still felt that CF impacted their decision to have children, and the impact of modulators on fertility and/or pregnancy was the most important SRH educational need, listed as “very” or “moderately” helpful by nearly 80% of respondents. This is especially important considering that the use of CFTR modulators during pregnancy is still being explored. Although early observations appear reassuring, there is an association with prenatal modulator use and cataract formation, and long-term child health outcomes remain unknown (16). A current, prospective multi-center observational study [Maternal and Fetal Outcomes in the Era of Modulators (MAYFLOWERS)] is evaluating outcomes in mothers taking modulators during pregnancy, as well as longer-term fetal and infant outcomes (17). Other work has and will be evaluating the use of modulators in pregnant CFTR carrier mothers of fetuses with CF to prevent CF complications (18, 19). While more data are needed, interest is certainly increasing in the CF community, and as knowledge of this potential off-label option disseminates, informed counseling will become even more important. Given the opinions and needs expressed in our study and limited existing data, routine counseling from specialists familiar with pregnancy in CF is a critical need moving forward.

Our study did have several limitations. First, although our response rate was reasonably high and participants had diverse sexuality, this was a single-center study in a large urban center, and all participants were English-speaking, and predominantly White race. Access to genetic counseling services is more available in urban areas compared to more rural communities, and perspectives in other populations may differ. Second, we intentionally chose to include all adults to capture diverse perspectives, and many individuals were past reproductive age. We did not observe a clear relationship between age and opinions on genetic counseling or SRH topics, but the numbers were small in the older cohort. Last, we chose to focus on patient perspectives on SRH and genetic counseling educational needs, but a detailed examination of solutions was not within the scope of this study. Certainly, many barriers to providing education exist in CF centers including costs and time, and further work will be needed to help delineate optimal strategies.

## Conclusions

There is a need for reproductive genetic counseling and education among adults with CF. Our study highlights many specific SRH knowledge gaps and educational needs, which have become critically important in the modern era of CF care. The current healthcare landscape of CF is changing with new, effective treatments, and CF care teams will need to adapt to support the evolving needs of CF patients.

## Supplementary Material

This is a list of supplementary files associated with this preprint. Click to download.

• BMCPulmonarySupplementCFGeneticcounselingsurvey.pdf

## Figures and Tables

**Table 1:** Demographics

Variable	Category	Value
Age	Median age (IQR), years	35 (29–41)
	Age range, years	19–84
Sex	Male	48 (41%)
	Female	67 (58%)
	Prefer not to answer	1 (1%)
Gender	Man	45 (39%)
	Woman	63 (55%)
	Nonbinary	6 (5%)
	Self describe: she/they	1 (1%)
	Prefer not to answer	1 (1%)
Sexuality	Heterosexual	91 (78%)
	Gay	1 (1%)
	Lesbian	2 (2%)
	Bisexual	14 (12%)
	Pansexual	3 (3%)
	Asexual	5 (4%)
	Queer	2 (2%)
	Self describe: gynosexual	1 (1%)
	Prefer not to answer	1 (1%)
Race	White	113 (97%)
	Middle Eastern/North African	2 (2%)
	Native American	4 (3%)
	East Asian	2 (2%)
	South Asian	1 (1%)
	Prefer not to answer	1 (1%)
Ethnicity	Hispanic/Latinx	3 (3%)
Modulator use	Vanzacaftor/tezacaftor/deutivacaftor	21 (18%)
	Ivacaftor	1 (1%)
	Tezacaftor/ivacaftor	1 (1%)
	Elexacaftor/tezacaftor/ivacaftor	84 (72%)
	Not on CFTR modulators	9 (8%)

CFTR=Cystic fibrosis transmembrane conductance regulator

**Table 2:** Respondents’ Family Composition and Plans

Currently have children	Yes	37 (32%)
	No	79 (68%)
	Median number (IQR) of children	1 (1–2)
	Number of children range	1–3
Planning future children	Yes	28 (24%)
	No	65 (56%)
	Undecided	23 (20%)
Did/does cystic fibrosis factor into your decision to have children?	Yes	74 (64%)
	No	40 (35%)
	Prefer not to answer	2 (2%)

**Table 3:** Categories/Subcategories and Exemplar Quotes

Category/subcategory	Example quotes (with participant ID and sex)
**Fertility status**	“Cannot have kids without intervention.” (ID# 031, male)
Possible male infertility due to congenital bilateral absence of the vas deferens	“I have never felt strongly about having kids and so being sterile made it feel like ’it wasn’t meant to be.” (ID# 119, male)
Female subfertilty	“I’ve heard people diagnosed with CF have a lower chance to have children…” (ID# 108, female)
Uncertainty about fertility status	“Don’t know if I can have kids and still need to get checked.” (ID# 037, male)
**Concerns and uncertainty with personal health**	“Though not the primary influencing reason, there’s a lot of complications and uncertainties that comes with having CF and living with someone with CF. Unfortunately, even with the improvement of treatments and the new CFTR modulators, a lot of the future still feels uncertain and if anything were to suddenly happen or take a turn for the worse, that would be devastating.” (ID# 111, male)
Prenatal/pregnancy health concerns	“I think also the extra toll pregnancy has on your body, in addition to the CF symptoms, can be intimidating.” (ID# 012, female)
Postnatal/parental health concerns	“As I’ve gotten older and spent time babysitting nieces and nephews, I’ve realized I just get sick so easily and kids are constantly sick. I’m not sure my health would allow it.” (ID# 089, male)
Mental health concerns	“It’s just really sad to me that I don’ think I am going to be able to have a child. Without CF, I feel pretty confident I could. But I don’t have a partner and I don’t want one, and I couldn’t do it without one. So… the concern is just that it’s hard to square that and it’s so omnipresent, even in the post modulator discussions.” (ID# 056, female)
**Ability to be present for children**	“I want to be able to live as full of a life as I can despite my Cystic Fibrosis so that my future children get to have their dad in their lives as long as possible” (ID# 121, male)
Limited life expectancy	“And can I be a reliable, present parent through their childhood? The possibility of becoming seriously ill-or dying-and leaving my partner to raise a child alone, or my child to grow up without a mother, weighs heavily on my mind.” (ID# 027, female)
Additional care burden related to a child	“CF influenced my decision to have no kids. It’s been hard enough to take care of myself, I can’t imagine being responsible for another life.” (ID# 095, female)
Non-biologic parenting options	“The last thing I want to share is how I felt I could be involved in my niece and nephews’ lives and have some sense of being part of children growing up. When I was young, a couple who did not have kids were involved in my life, and I felt they embodied the saying that it takes a village to raise a child. I felt I could be part of that village without having my own children.” (ID# 005, female)
**Concerns about children’s health/not wanting to pass on CF**	“I do not want my child to grow up with CF; it is challenging to live with mentally and physically. I do not want them to go through that struggle too.” (ID# 092, female)
Reasons for not wanting to pass on CF	“I think it is genetically irresponsible to have children with cystic fibrosis. I don’t know if any of you have noticed but cystic fibrosis is both a horrible disease as well as horribly expensive disease” (ID# 022, female)
Carrier screening for partners	“When we decided to start trying to get pregnant my husband had not yet been tested to see if he was a CF carrier. We tested my husband while we were pregnant with our first child. Abortion was not an option for us, but we wanted to be prepared medically if our baby was going to be born with CF. My husband tested negative and both our children were born without CF.” (ID# 059, female)
**Financial considerations with family planning**	“Cost of additional fertility treatments and planning post-pregnancy are complicated by CF.” (ID# 079, male)
Financial and emotional challenges with in vitro fertilization/assisted reproductive technologies	“Alternative options such as IVF were very expensive. I wanted kids but lots of challenges and financial barriers.” (ID# 101, male)
Cost of personal and additional healthcare	“Unless healthcare magically becomes free in my lifetime, I will never be comfortable knowing that there would be times I would need to choose between healthcare for myself or healthcare for my child.” (ID# 054, female)
Children contributing to financial insecurity	“Kids are a huge expense, and I’m already doubtful of my chances of having financial stability given the current economy and political situation. I want to give myself every chance of financial wellbeing, which to me means no kids.” (ID# 076, female)
**Advancements in treatments create reproductive opportunities**	“After modulators–and with a drastic change in my life expectancy–we could change our minds and are currently pursuing IVF.” (ID# 116, male)
Modulators improving health, so pregnancy is an option	“Prior to going on a modulator, I was unsure if my CF would be stable enough to have children. I still wonder this sometimes, but the modulator has decreased the worry.” (ID# 110, female)
**Late diagnosis**	“I was diagnosis when I was 71 years old. TOOOOOO old for children.” (ID# 072, male)
CF may have impacted fertility before known diagnosis	“I was past reproductive age when diagnosed with CF. I did have infertility though. I really wanted to have a child and started trying to get pregnant at age 30. After a year and a half of timing intercourse with basal body temperature efforts to predict ovulation, my husband’s sperm was tested and was normal. I had always suspected I would have difficulty getting pregnant because I had never been pregnant before.” (ID# 034, female)
**General/other reproductive health considerations**	“One concern I have, is I’ve read CF patients can have lower hormonal levels. I’d be interested to have more routine screening on that. I don’t really know what “normal” amounts of hormones feel like, so it’s hard to know if I’m off. Sometimes I wonder about it.” (ID# 070, male)
How CF affects menopause	“Along with every other menopausal woman in the world today my biggest concerns are that there is so little information and help regarding perimenopause and menopause. I have found the support I need but you have to be a strong personal advocate.” (ID# 025, female)
Sex hormones and hormonal birth control	“I’ve never had a discussion on sex hormones and how those, plus oral contraceptives can affect CF. From a medical standpoint, that would be very interesting to learn about and should be discussed with patients!” (ID# 038, female)
**Lack of informed discussions with providers**	“Are there obgyns who understand CF? because most GPs I’ve seen know nothing and just assume all my health concerns are CF related” (ID# 011, female)
Gynecologist/primary care provider doesn’t know about CF	“My gynecologists have never known how to discuss my reproductive health as it relates to CF” (ID# 002, female)
CF providers don’t know/talk about sexual and reproductive health	“I have never had a CF provider discuss reproductive healthcare with me ever. I feel very certain of my desire to never be pregnant and never have a kid, but I still feel like providers should be discussing these things with their adult patients. A huge problem I had with my pediatric CF team, who were wonderful, was that they never talked about what I could expect from my adult life living with CF. This was before modulators, so obviously a lot has changed, but I still feel like at my appointments the focus is on the immediate short term.” (ID# 076, female)

CF=Cystic fibrosis; CFTR=Cystic fibrosis transmembrane conductance regulator; IVF = In vitro fertilization

## Data Availability

The data used and analyzed during the current study are available from the corresponding author on reasonable request.

## Abbreviations

CF: Cystic fibrosis
CBAVD: Congenital bilateral absence of the vas deferens
pwCF: People with CF
ART: Assisted reproductive technologies
CFTR: Cystic fibrosis transmembrane conductance regulator
SRH: Sexual and reproductive health
PGT-M: Preimplantation genetic testing
IVF: In vitro fertilization
